# *Garcinia kola* (Heckel) and *Alchornea cordifolia* (Schumach. & Thonn.) Müll. Arg. from Cameroon possess potential antisalmonellal and antioxidant properties

**DOI:** 10.1371/journal.pone.0237076

**Published:** 2020-08-04

**Authors:** Fred Djague, Paul Keilah Lunga, Kouipou Rufin Marie Toghueo, Dongmo Yanick Kevin Melogmo, Boyom Fabrice Fekam

**Affiliations:** Antimicrobial and Biocontrol Agents Unit (AmBcAU), Laboratory for Phytobiochemistry and Medicinal Plants Studies, Department of Biochemistry, Faculty of Science, University of Yaoundé I, Yaoundé, Cameroon; Universite d'Orleans, FRANCE

## Abstract

Drug resistant *Salmonella* species and shortcomings related to current drugs stress the urgent need to search for new antimicrobial agents to control salmonellosis. This study investigated the antisalmonellal and antioxidant potentials of methanolic and hydro-ethanolic extracts of *Garcinia kola* and *Alchornea cordifolia* as potential sources of drugs to control *Salmonella* species and to reduce related oxidative stress. The antisalmonellal activity was assessed using the broth microdilution, membrane destabilization and time-kill kinetic assays. While, the DPPH, ABTS and FRAP assays were used for the determination of the antioxidant activities. The minimum inhibitory concentrations ranged from 125 to 1000 μg/mL, with the methanolic root extract of *G*. *kola* being the most active. The time kill kinetic assay revealed a concentration-dependent bacteriostatic activity for promising extracts. Potent extracts from *G*. *kola* showed the ability to destabilize *S*. *typhi* outer membrane, with the methanolic root extract presenting the highest activity; two-fold higher than those of polymyxin B tested as reference. In addition, this methanolic root extract of *G*. *kola* also provoked nucleotide leakage in a concentration-dependent manner. From the antioxidant assays, the hydro-ethanolic extract from the stem bark of *A*. *cordifolia* presented significant activities comparable to that of Vitamin C. The methanolic root extract of *G*. *kola* also presented appreciable antioxidant activities, though less than that of *A*. *cordifolia*. Overall, the phytochemical screening of active extracts revealed the presence of anthocyanins, flavonoids, glycosides, phenols, tannins, triterpenoids and steroids. These results provide evidence of the antibacterial potential of *G*. *kola* and offer great perspectives in a possible standardisation of an antisalmonellal phytomedicine.

## Introduction

Salmonellosis is an infectious disease caused by *S*. *enterica*, and *S*. *bongori* [1]. Historically, it has been clinically categorized as typhoidal and non-typhoidal [2]. The former category is due to the human host-restricted *S*. *enterica serovars typhi*, *paratyphi A*, *paratyphi B* and certain strains of *S*. *paratyphi C* that cause typhoid and paratyphoid fevers, referred collectively as enteric fevers. The latter is due to ubiquitous non-typhoidal *Salmonellae* (NTS) such as *S*. *enterica serovar typhimurium* and *S*. *enterica serovar enteritidis* having broader host range and predominantly causing self-limiting gastroenteritis in animals and humans [3]. *Salmonella* bacteria are widely distributed in domestic and wild animals.

Transmission is generally via the faecal-oral route, by either the consumption of contaminated food or water, person-to-person contact, or from direct contact with infected animals [4]. Salmonellosis is manifested in several different ways and both sex are affected equally [5]. The predominant symptom for typhoidal salmonellosis is fever. The temperature rises gradually during the first week of the illness and reaches a plateau of 39 to 40°C the following week. Other common symptoms include: abdominal pain, diarrhea, constipation, loss of appetite, headache etc. Some patients experience distinctive rash on their chest and abdomen, which is rose-coloured and flat, while others develop hepatosplenomegaly [6]. Acute gastroenteritis is the most common presentation of NTS infection. Typical symptoms include nausea, and/or vomiting. Fever, abdominal cramps and bloody diarrhea may also be reported [7].

Salmonellosis remains a major public health problem worldwide [1,8]. It is the second most commonly reported foodborne infection in the European Union and in the United States [9]. In Africa, recent investigation showed that the relative proportion of bacteremia due to invasive *Salmonella* infections has increased dramatically [10]. Typhoidal salmonellosis continue to be a significant contributor to global morbidity and mortality, with an estimated 17 million illnesses worldwide and approximately 178,000 deaths each year as of 2015 [8]. Besides enteric fever, non-typhoidal salmonellosis also represent a considerable burden in both developing and developed countries [11]. Each year, an estimated 94 million cases and approximately 155 000 deaths occur due to gastroenteritis worldwide [12]. There are several drugs (Amoxicillin, Ampicillin, Ciprofloxacin, Cefotaxime, Ceftriaxone, etc.) used to fight this infection [13]. However, due to many reasons including the extensive and inappropriate use of the same antimicrobials, delay in diagnosis or poor hygiene conditions, increase in multidrug resistant *Salmonella* species has been reported, restricting the therapeutic options [14].

Apart from causing salmonellosis, Rastaldo *et al*. [15] reported that the entrance of *Salmonella* equally causes the production of superoxide and nitric oxide which react together to form peroxynitrite a strong biological oxidant, leading to increase levels of oxidative species (hydrogen peroxide, and hydroxyl radical) and oxidative stress [16]. Stress is at the origin of other human illnesses like sickle cell diseases, artherosclerosis, Parkinson’s disease, heart failure, myocardial infarction, Alzheimer’s disease etc. [17]. Oxidative stress can be prevented or delayed by using antioxidant agents. However, beside their unavailability, high cost and side effects, frequently used synthetic antioxidants such as Butylated Hydroxyl Anizole (BHA) and Butylated Hydroxyl Toluene (BHT) have been proven to be neurotoxic, hepatotoxic, and carcinogenic [18]. Consequently, there is need to search for complementary and alternative sources of medicines.

*Garcinia kola* Heckel is a dicotyledonous plant belonging to the family of Clusiaceae [19]. It is mostly found in lowland forests of India, Indochina, Indonesia, and Brazil [20]. It has been recognized as an indigenous medicinal plant found in the tropical rain forest of Central and Western Africa, especially Benin, Sierra Leone, Democratic Republic of Congo, Ivory Coast, Gabon, Ghana, Liberia, Nigeria, Senegal and Cameroon, where it is one of the most valued tree [21]. Known as bitter cola, the fruits of *G*. *kola*, is a well-recognized aphrodisiac, and a decoction of the leaves and stem bark is traditionally used in the treatment of typhoid fever in Cameroon. *Alchornea cordifolia* Schum. & Thonn. on the other hand, belongs to the family of Euphorbiaceae and occurs mainly in west to central Africa in countries as Congo, Ivory Coast, Nigeria, Ghana and Cameroon. [22]. It is called Bambami in Hausa (Nigeria), Mabounji in the southwest region of Cameroon, Arbre de djeman in French and commonly Christmas bush or dove wood in English. It is commonly used throughout its areas of distribution as a remedy for skin diseases, abdominal pains and vomiting.

Previous studies [23–25] reported the antibacterial activity of *G*. *kola*. Despite the few works done on the antisalmonellal activity of *G*. *kol*a, investigations related to the possible mechanisms of action of *G*. *kola* extracts are still not very much explored. In addition, most of the biological activities have been performed on seeds; and reports of these activities on the roots, stem barks and leaves of *G*. *kol*a are lacking. More so, most of the activities are evaluated by using preliminary and inconclusive tests such as the agar disc or well diffusion methods. For *A*. *cordifolia*, despite the works done on this plant, its hydro-ethanolic extract has not been used. Thus, the aim of the present study was to evaluate the antisalmonellal and antioxidant properties of the leaves, roots and stem barks of *G*. *kola* and *A*. *cordifolia* and their possible antimicrobial modes of action.

## Materials and methods

### Plant materials and extraction

Fresh parts of a specimen of both *G*. *kola* and *A*. *cordifolia* were harvested from Kumba, Southwest Region of Cameroon in March 2018. These parts included the leaves, roots and stem bark of *G*. *kola* and the leaves, stem bark and twigs of *A*. *cordifolia*. Botanical identification was done by Mr Nana Victor at the Cameroon National Herbarium in Yaoundé, where a voucher specimen was deposited under the reference number 41604HNC and 29705HNC respectively for *G*. *kola and A*. *cordifolia*. No permission was necessary for sample collection. The fresh plant materials were later washed separately with fresh water to remove dirt and other contaminants, shade-dried and ground to fine powders. The powders were macerated in hydro-ethanol (1:4 v/v) and absolute methanol for 72 hours accompanied by occasional shaking and stirring. The solutions were sieved using a hydrophilic cotton and filtered using Whatman filter paper N^0^ 1 after which the filtrates were concentrated using a rotary evaporator (BÜCHI 461) at 65ºC (for hydro-ethanol) and at 60ºC (for methanol) under reduced pressure till dryness to obtain the crude extract. The extracts were preserved in sterile bottles and conserved at 4 ºC for further experiments.

### *In vitro* antisalmonellal assay

#### Microbial species and culture media

Three *Salmonella* species were used in this study, one strain (*S*. *typhi*) from the ‘‘Centre Hospitalier Universitaire” (CHU) of Yaounde-Cameroon and two clinical isolates (*S*. *typhimurium* and *S*. *enteritidis*) from ‘‘Centre Pasteur” of Cameroon (CPC). Mueller Hinton Agar (MHA) was used to revive the bacteria while Mueller Hinton Broth was used for the antisalmonellal assay. Salmonella-Shigella Agar (SS agar) was used for the time kill kinetic assay.

#### Determination of MIC and MBC

The minimum inhibitory concentration (MIC) and minimum bactericidal concentration (MBC) were determined by the broth micro-dilution method, using the ρ-iodonitrotetrazolium-Chloride (INT) based assay as previously reported by Lunga *et al*. [26]. Briefly, 196 μL of Muller Hinton broth (MHB) were introduced into the wells of the first line followed by 4 μL of each extract (initially prepared at 100 mg/ml in 100% DMSO). Serial two-fold dilutions were performed and 100 μL of the bacterial suspension (1.5 x10^6^ CFU/mL) was added into each well. The final concentration ranged from 1000 to 15.625 μg/mL for the extracts and from 5 to 0.0195 μg/mL for the positive control (Ciprofloxacin and Polymyxin B).The plates were incubated at 37°C for 24 hours after which 50 μL of 0.2 mg/mL INT solution was added and further incubated at 37°C for 30 min. MICs were determined as the lowest sample concentration at which no visible colour change was observed. Wells containing MHB and bacteria constituted the negative control while the sterility control contained MHB alone. The final concentration of DMSO was at most 1% and preliminary test did not inhibit bacterial growth

The MBC was determined by transferring 50 μL of INT-free aliquots from the wells with no colour change into 150 μL of freshly prepared MHB. These preparations were further incubated at 37°C for 48 hours and revealed as above to obtain the MBC. All tests were carried out in triplicates.

### Phytochemical screening

The presence of major phytochemical classes of compounds such as Alkaloids, Anthocyanins, Flavonoids, Glycosides, Phenols, Quinones, Saponins, Sterols, Tannins and Triterpenoids were qualitatively evaluated in the plant extracts according to previously described methods [27].

### Evaluation of possible mode of action

#### The outer membrane permeability (OMP) assay

This was determined according to the method described by Hao *et al*. [28], with some modifications. In a 96 well micro-plate, concentrations of MIC, 2MIC, and 4MIC of each extract in triplicate were prepared by two-fold serial dilution. One hundred microliters (100μL) of *S*. *typhi* suspension (1.5 x 10^6^ CFU/mL) were added and the plate incubated at 37°C for 24h. The optical densities were read at 405 nm (wavelength at which the complex form between Lipopolysaccharides and membrane stabilizing divalent cations absorbs) and the percentage destabilization was calculated using the formula below.

$$\%D=\frac{Ao-As}{Ao}x\;100$$

Where ***%D***: *Percentage Destabilization;* ***Ao***: *Absorbance of negative control;* ***As***: *absorbance of test samples*.

Polymyxin B was used as the positive control and was equally prepared at MIC (0.0781 μg/mL), 2MIC (0.1562 μg/mL), and 4MIC (0.2124 μg/mL). MHB and the bacteria constituted the negative control while the sterility control contained MHB alone. Wells containing MHB and the extracts alone constituted the blank. A graph of %D against extract concentration was plotted in order to determine the concentration which causes maximum destabilization.

#### Nucleotide leakage

This was determined according to the method described by Tang *et al*. [29] with slight modifications. Briefly, an overnight culture of *S*. *typhi* was washed in sterile physiologic water (2 mL of 0.9% NaCl) and the resulting solution centrifuged at 10 000 rpm for 10 min. The supernatant was discarded, and the resulting pellet was re-suspended in 10 mM PBS (pH 7.4) and the turbidity adjusted to 0.5 McFarland (1.5x10^8^ CFU/mL). In a 96 well micro-plate, 100 μL of the bacterial suspension were added to 100 μL of MHB containing the methanolic root extract of *G*. *kola* (GKRM) at MIC, 2MIC, 4MIC (initially prepared in 10 mM PBS (pH 7.4)). The plate was incubated at 37°C for different times (0, 2, 4, 6, 8, 12 and 24h). Following each incubation period, the cell suspension was centrifuged at 10 000 rpm for 10 min, the supernatant appropriately diluted and the optical densities were recorded at 260 nm. The test was performed in triplicate and simultaneously for positive (ciprofloxacin), negative (PBS + cell suspension), sterility control (MHB alone) and blank (MHB + extract). The optical densities were plotted against the different times to determine the time dependent degree of leakage of the different extract concentrations.

#### Time-kill kinetics test

The time kill kinetic of the *G*. *kola* root methanol (GKRM) extract was performed according to the method described by Tsuji *et al*. [30]. Here, extract concentrations of MIC, 2MIC, 4MIC and 8MIC were prepared by serial two-fold dilution in a 96 well micro-plate. One hundred microliters (100 μL) of *S*. *typhi* suspension (1.5 x 10^6^ CFU/mL) were added and the plate incubated at 37°C for different time intervals (0, 1, 2, 4, 6, 8, 12, and 24 h). Following each incubation period, the cell suspensions were appropriately diluted (in NaCl 0.9%) and the resulting solution sub-cultured on SS agar plates for further 24 h at 37°C. Ciprofloxacin was used as positive control while the bacteria incubated with MHB was used as growth control. The test was performed in triplicate and results were presented as Mean ±SD.

### *In vitro* antioxidant activity

#### DPPH radical scavenging assay

This was performed according to the method described by Scherer and Godoy [31] with slight modifications. Briefly, the extracts were first diluted to obtain final extract concentrations of 2000, 1000, 500, 250, 125, 62.5, 31.25, and 15.625 μg/mL in a 96 well micro-plate. Twenty-five microliters (25 μL) of each dilution was introduced into a new micro-plate and 75 μL of 0.02% DPPH added to obtain final concentrations of 500, 250, 125, 62.5, 31.25, 15.625, 7.813 and 3.906 μg/mL. The reaction mixtures were kept in the dark at room temperature for 30 min after which the absorbance was measured at 517 nm against the blank (DPPH in methanol). L-ascorbic acid was used as positive control and was treated in the same conditions as the extracts with final concentrations of 25, 12.5, 6.25, 3.125, 1.563, 0.781, 0.391 and 0.195 μg/mL. The assay was performed in triplicate.

The percentage (%) radical scavenging activities of the plant extracts were calculated using the following formula; from which other parameters like the radical scavenging activity 50 (RSA_50_), effective concentration 50 (EC_50_), and antiradical power (ARP) were deduced.

$$\%\;RSA=\frac{Ao-As}{Ao}x\;100$$

*Where*, ***RSA**: Radical Scavenging Activity; **Ao:** Absorbance of the blank (DPPH + methanol); **As:** Absorbance of DPPH radical + plant extract*.

#### ABTS radical scavenging assay

It was performed according to the method described by Re *et al*. [32] with slight modifications. In a 96 well micro-plate, the assay was performed at the same extract concentrations as above, using the same positive control. The absorbance of ABTS radical after 30 min incubation was read at 734 nm. The assay was performed in triplicate.

The percentage (%) radical scavenging activities of the plant extracts were calculated and from it, the same parameters were deduced as for the DPPH assay.

#### Ferric ion reducing antioxidant power (FRAP) assay

The assay was performed according to the method described by Benzie *et al*. [33] with slight modifications. Briefly, the extracts were first dissolved as for the DPPH and ABTS assays. 25 μL from each dilution were added to 25 μL of 1.2 mg/mL Fe^3+^ solution in a new micro-plate. The plates were pre-incubated for 15 min at ambient temperature. Fifty microlitres (50 μL) of 0.2% ortho-phenanthroline solution was added to obtain final extract concentrations of 500, 250, 125, 62.5, 31.25, 15.625, 7.8125 and 3.90625 μg/mL. The reaction mixtures were further incubated for 20 min at ambient temperature after which the absorbance was measured at 505 nm using a 96 well micro-plate reader (Infinite M200 (TECAN) against the blank (25 μL methanol + 25 μL Fe^3+^ + 50 μL ortho-phenanthroline). Hydroxylamine was used as positive control and was treated in the same way as the extracts with final concentrations of 6.6, 3.3, 1.65, 0.825, 0.413, 0.206, 0.103 and 0 μg/mL. The assay was performed in triplicate.

From a concentration-activity curve of NH_2_OH used as standard, the optical densities of the test wells were projected, and the results expressed quantitatively as μg equivalent NH_2_OH/g of extracts.

### Statistical analysis

Where appropriate, the data were subjected to one-way analysis of variance (ANOVA) and results were presented as the Mean ± SD of the replicated values. Significance differences for multiple comparisons were determined by Waller-Duncan Post Hoc test at p ≤ 0.05 using the Statistical Package for the Social Sciences (SPSS, version 16.0) program.

## Results

### Antisalmonellal activities of the extracts

The microbial growth inhibition capacities of the extracts were assessed based on their minimum inhibitory concentrations (MICs) and bactericidal concentrations (MBCs) (Table 1).

**Table 1:** Minimum inhibitory (MIC) and bactericidal (MBC) concentrations (μg/mL) of the methanolic and hydro-ethanolic extracts of *G*. *kola* and *A*. *Cordifolia*.

Sample	Parameter	Microorganisms		
		STM	SE	ST
**GKLM**	MIC	/	/	250
	MBC	─	─	/
**GKRM**	MIC	250	250	125
	MBC	/	/	/
**GKRH**	MIC	500	250	250
	MBC	/	/	/
**GKSM**	MIC	500	500	250
	MBC	/	/	/
**GKSH**	MIC	/	250	125
	MBC	─	/	/
**ACSH**	MIC	1000	/	1000
	MBC	/	─	/
**Ciprofloxacin**	MIC	0.078	0.156	0.039

STM: *Salmonella typhimurium*; SE: *Salmonella enteritidis*; ST: *Salmonella typhi*; / MIC or MBC ˃ 1000 μg/mL; ─: Not tested, GKLM: *G*. *kola* Leaf MeOH; GKRM: *G*. *kola* roots MeOH; GKRH: *G*. *kola* roots H_2_O/EtOH; GKSM: *G*. *kola* stem MeOH, GKSH: *G*. *kola* stem H_2_O/EtOH; ACSH: *A*. *cordifolia* stem H_2_O/EtOH.

The MIC values ranged from 125 to 500 μg/mL. *G*. *kola* extracts were generally more active (MIC ranging from 125 to 500 μg/mL) than those of *A*. *cordifolia* (MIC 500 μg/mL). Of all *G*. *kola* extracts, both the hydro-ethanolic stem bark and its methanolic leaf extracts were not active against *S*. *typhimurium* while the methanolic leaf extract alone was inactive against *S*. *enteritidis* at the tested concentrations. The results also showed that *S*. *typhi* was the most susceptible of the three serovars (MIC of 125μg/mL with the methanolic root and hydro-ethanolic stem bark extracts of *G*. *kola* and 250 μg/ml with other *G*. *kola* extracts).

### Phytochemical composition of extracts

Phytochemical screening revealed the presence of Anthocyanins, Flavonoids, Glycosides, Phenols, Tannins, Triterpenoids and Steroids (Table 2). The methanolic leaf extract of *G*. *kola* and the Hydro-ethanolic stem extract of *A*. *cordifolia* were the most complex in phytochemical groups, though being the least antisalmonellal. The methanolic root extract of *G*. *kola*, with the best antibacterial activity, was the least complex in phytochemical groups of compounds, differing from its hydro-ethanolic extract by the presence of glycosides and terpenoids on one hand, and the absence of flavonoids on the other hand in the latter. This may suggest the central role played by flavonoids in attributing activity to this plant.

**Table 2:** Phytochemical composition of *G*. *kola* and *A*. *cordifolia* extracts.

Extracts	Phytochemical classes of compounds									
	Alkaloids	Anthocyanins	Flavonoids	Glycosides	Phenols	Quinones	Tannins	Triterpenes	Saponins	Steroids
**GKLM.**	**-**	**+**	**+**	**+**	**+**	**-**	**-**	**+**	**-**	**+**
**GKRM**	**-**	**-**	**+**	**-**	**+**	**-**	**+**	**-**	**-**	**-**
**GKRH**	**-**	**-**	**-**	**+**	**+**	**-**	**+**	**+**	**-**	**-**
**GKSM**	**-**	**-**	**+**	**-**	**+**	**-**	**+**	**-**	**-**	**-**
**GKSH**	**-**	**-**	**+**	**-**	**+**	**-**	**+**	**+**	**-**	**-**
**ACSH**	**-**	**+**	**+**	**+**	**+**	**-**	**+**	**-**	**-**	**-**

Abbreviation: +: presence of phytochemical; -: absence of phytochemical; GKLM: *G*. *kola* Leaf MeOH; GKRM: *G*. *kola* roots MeOH; GKRH: *G*. *kola* roots H_2_O/EtOH; GKSM: *G*. *kola* stem MeOH, GKSH: *G*. *kola* stem H_2_O/EtOH; ACSH: *A*. *cordifolia* stem H_2_O/EtOH.

### Effect of extracts on outer membrane permeability of *S*. *typhi*

**Fig 1:**
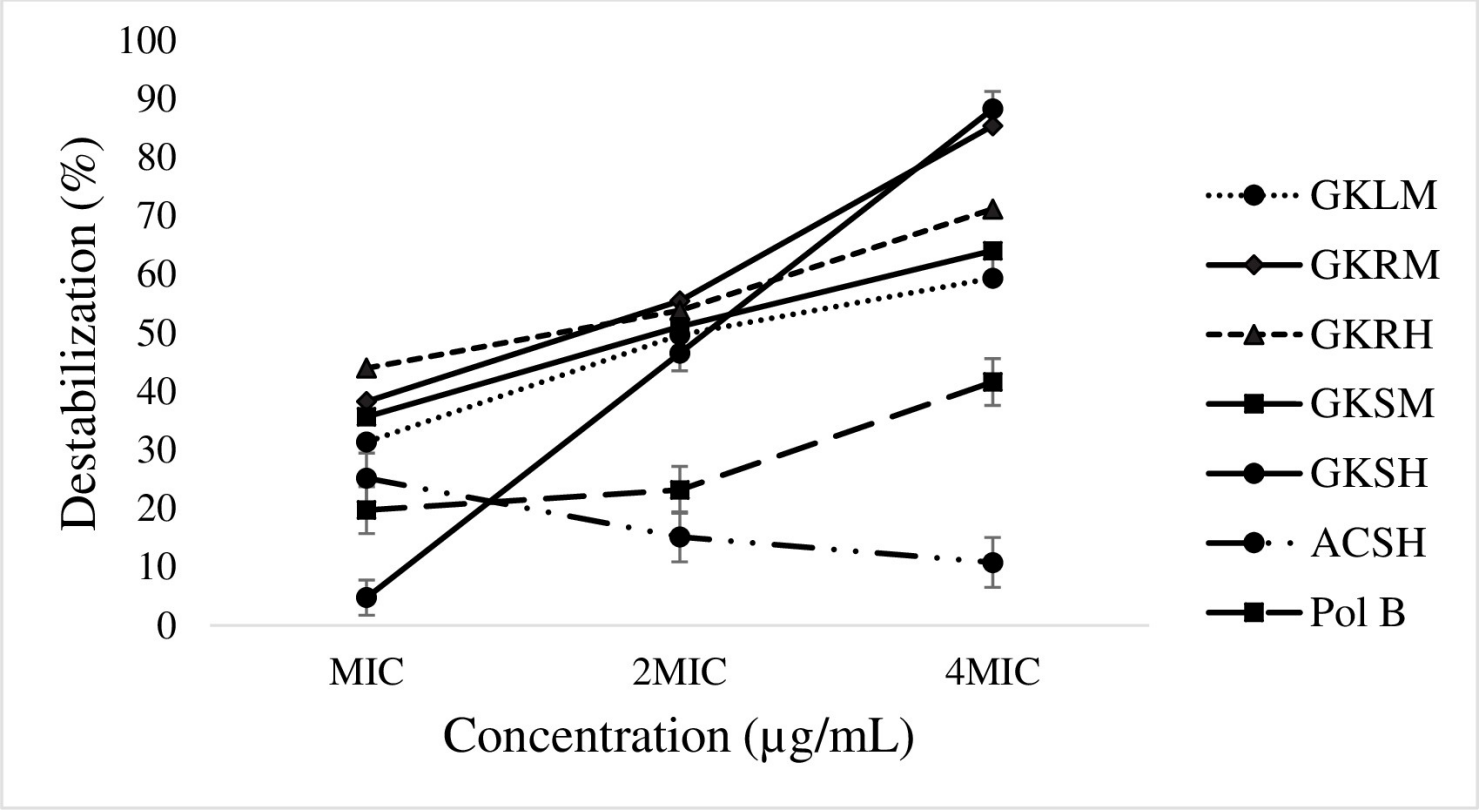
Percentage destabilisation effect of extracts on *S*. *typhi* outer membrane as a function of concentration. Pol B: Polymyxin B; GKRM: *G*. *kola* roots methanol; GKRH: *G*. *kola* roots hydro-ethanol; GKSM: *G*. *kola* stem bark methanol; GKSH: *G*. *kola* stem bark hydro-ethanol; GKLM: *G*. *kola* leaf methanol; ACSH: *A*. *cordifolia* stem bark hydro-ethanol.

The ability of the extracts to permeabilize the outer membrane of *S*. *typhi* was evaluated over a period of 24 h at three different concentrations (MIC, 2MIC and 4MIC). Fig 1 shows that, apart from the hydro-ethanolic stem bark extract of *A*. *cordifolia*, all the other extracts from *G*. *kola* presented the same trend as that of Polymyxin B, on the destabilization of *S*. *typhi* outer membrane. The results showed that the percentage permeabilization was concentration dependent. Interestingly, the destabilization percentage of the extracts of *G*. *kola* were generally significantly greater (p≤0.05) than those of Polymyxin B at their corresponding concentrations. The root methanolic extract showed the highest effect, closely followed by its hydro-ethanolic extract, then the stem and leaf methanolic extracts. The hydro-ethanolic stem bark extract of *G*. *kola* presented sky-rocketing activities at high concentrations; showing the highest effect at 4 MIC.

### Effect of *G*. *kola* methanolic root extract concentration on *S*. *typhi* nucleotide leakage with time

**Fig 2:**
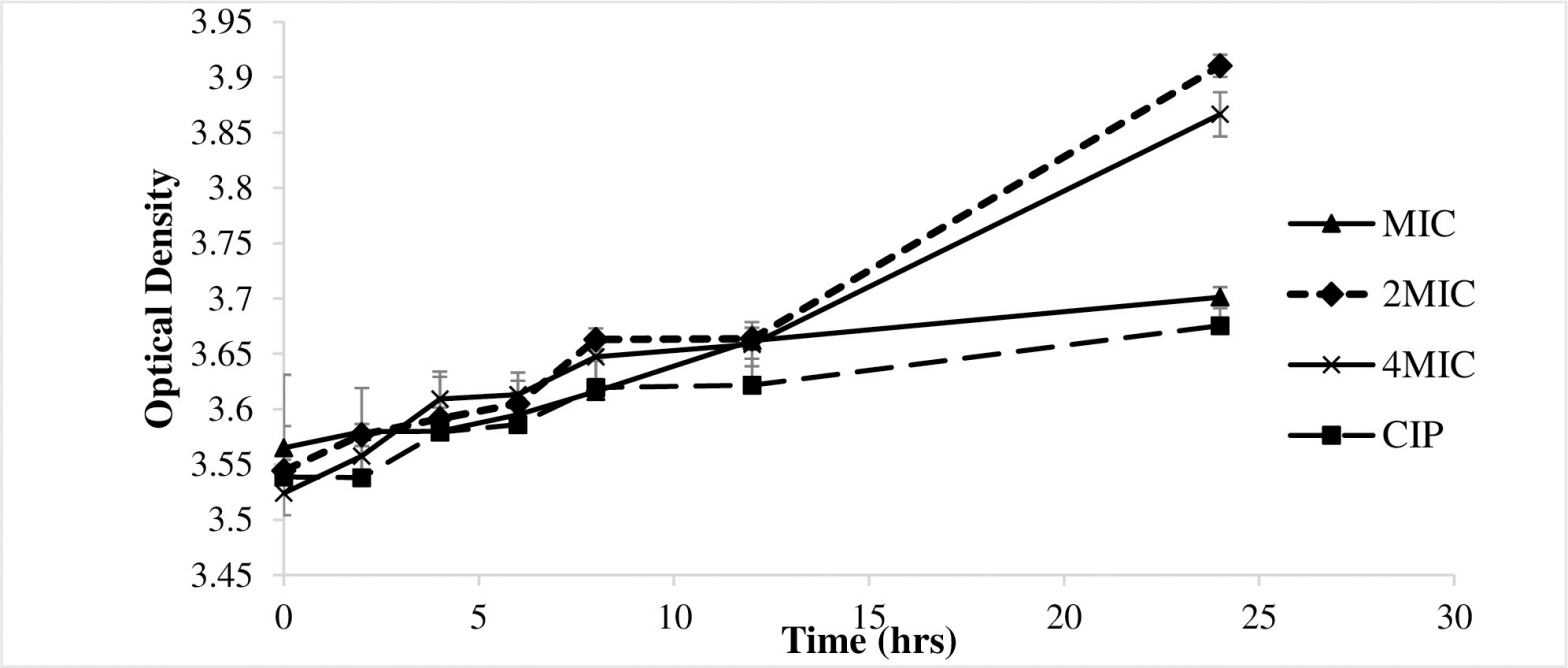
Effect of the methanolic root extract of *G*. *kola* on nucleotide leakage. CIP: Ciprofloxacin; ST: *Salmonella typhi*.

Being the most active antisalmonellal extract, the methanolic root extract of *G*. *kola* (GKRM) was further evaluated for its ability to permeabilize the cytoplasmic membrane. The results showed that GKRM extract caused leakage of nucleotides (DNA and RNA), as revealed by the increase in absorbance of the supernatant at 260 nm with time (Fig 2). This indicated that nucleotides were released outside the cell. The extract presented high nucleotide leakage effect at 2MIC, not significantly different (p≤0.05) from that of 4MIC; both of which were significantly different from the effect at MIC, the latter being similar to the effect of ciprofloxacin, the reference mechanistic antibiotic.

### The time-Kill kinetics effect of *G*. *kola* methanolic root extract against *S*. *typhi*

**Fig 3:**
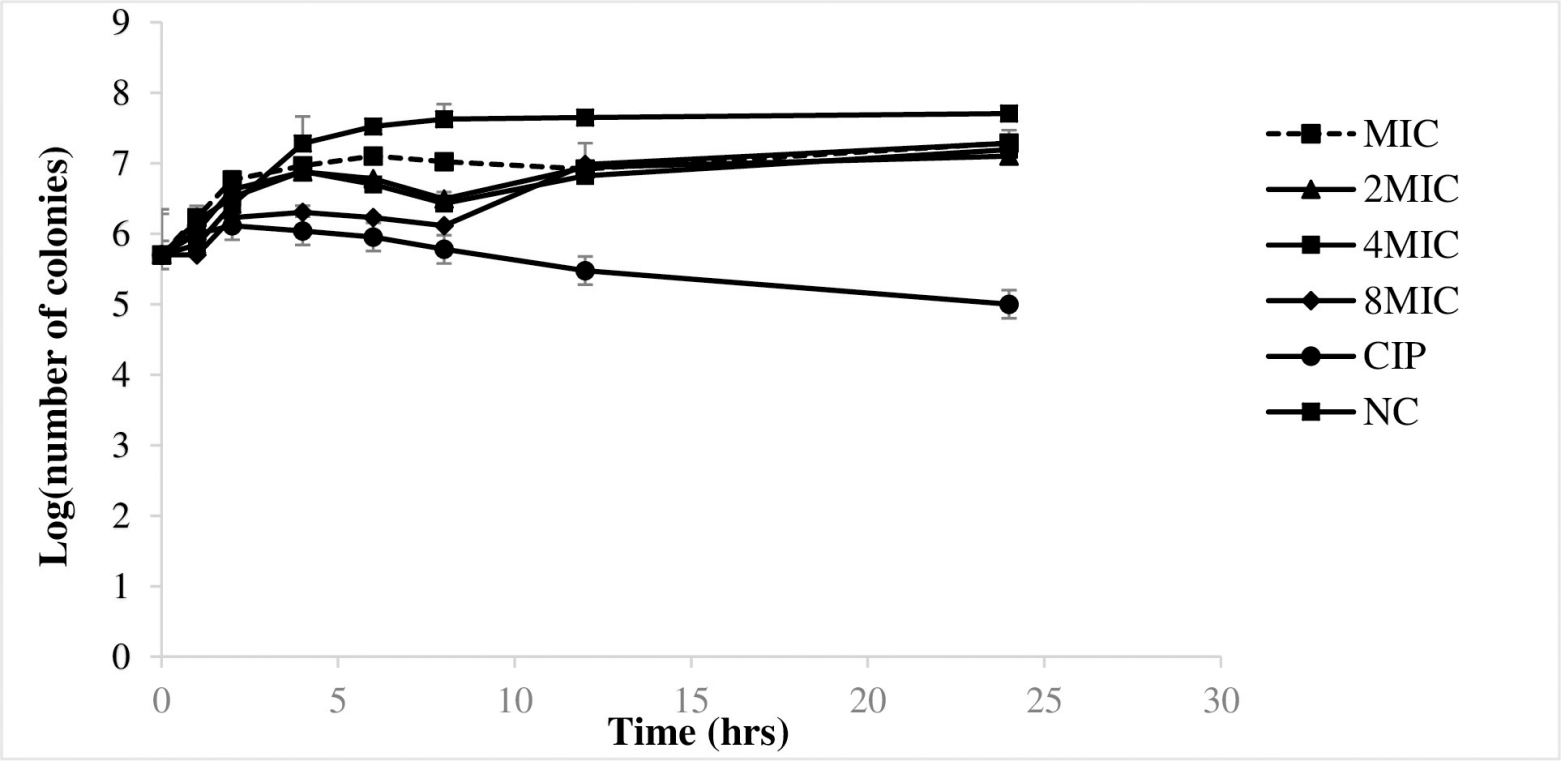
Time-Kill curve of *S*. *typhi* following exposure to various concentrations of methanolic roots extract of *G*. *kola*. CIP: Ciprofloxacin; NC: negative control; MIC: minimum inhibitory concentration.

All concentrations of the methanolic root extract of *G*. *kola* inhibited the growth cycle curve when compared to the negative control (Fig 3). There was an initial increase in the number of viable cells over the first 4 hours, followed by a reduction up to the 8^th^ h and a gradual rise to the 24^th^ h. The activities of the extract at 2MIC and 4MIC were not significantly different (p˃0.05), while at the MIC value, the extract concentration was insufficient to significantly reduce bacterial growth. The inhibitory activity (through membrane destabilization) was pronounced during the first 8 hours. After 8 hours (resurface or re-emergent time), the bacterial regained its normal growth cycle, indicating that the extract exhibited a concentration-dependent bacteriostatic effect on *S*. *typhi* as opposed to the bactericidal effect exhibited by ciprofloxacin (continuous declining trend of the growth curve).

### *In vitro* antioxidant potentials of the extract

The DPPH and ABTS assays revealed that the extracts exhibited good free radical scavenging activities (Tables 3 and 4). The hydro-ethanolic stem bark extract of *A*. *cordifolia* exhibited the highest radical scavenging activity (12.606 μg/mL and 1.330 μg/mL for DPPH and ABTS respectively), very close to that of Vitamin C (8.926 μg/mL and 2.710 ± 0.080 μg/mL). The methanolic root extract of *G*. *kola* presented appreciable antioxidant activities though less than that of *A*. *cordifolia*, the most antioxidant. The EC_50_ and ARP showed the same trend as the RSA_50_.

**Table 3:** 50% Radical scavenging activity (RSA_50_), 50% efficient concentration (EC_50_) and anti-radical power against DPPH radical.

Extract	RSA_50_(μg/mL)	EC_50_ x 10^3^(μg/mL)	ARP x 10^−5^
**GKLM**	22.183 ± 2.135^c^	1.478 ± 0.107^c^	67.659 ± 8.765^d^
**GKRM**	73.073 ± 5.485^e^	4.871 ± 0.274^e^	20.530 ± 2.068^b^
**GKRH**	126.400 ± 4.840^f^	8.427 ± 0.242^f^	11.867 ± 0.607^a^
**GKSM**	57.190 ± 3.830^d^	3.813 ± 0.191^d^	26.226 ± 2.353^c^
**GKSH**	17.920 ± 2.850^bc^	1.195 ± 0.143^bc^	83.682 ± 18.32^e^
**ACSH**	12.606 ± 0.415^ab^	0.840 ± 0.021^ab^	119.048 ± 5.249^f^
**VitC**	8.926 ± 1.065^a^	0.595 ± 0.053^a^	168.067 ± 27.18^g^

Along the line, values carrying the same letter superscripts are not significantly different (P>0.05), Waller Duncan; RSA_50_, 50% Radical scavenging activity; EC_50_ = 50% Efficient Concentration; ARP, Antiradical Power, GKLM: *G*. *kola* Leaf MeOH; GKRM: *G*. *kola* roots MeOH; GKRH: *G*. *kola* roots H_2_O/EtOH; GKSM: *G*. *kola* stem MeOH, GKSH: *G*. *kola* stem H_2_O/EtOH; ACSH: *A*. *cordifolia* stem H_2_O/EtOH.

**Table 4:** 50% Radical scavenging activity (RSA_50_) and 50% efficient concentration (EC_50_) and anti-radical power against ABTS radical.

Extract	RSA_50_(μg/mL)	EC_50_ x 10^3^(μg/mL)	ARP x 10^−5^
**GKLM**	17.860 ± 1.010^e^	16.237 ± 0.918^e^	6.172 ± 0.349^a^
**GKRM**	8.786 ± 3.305^b^	7.988 ± 3.005^b^	13.895 ± 5.615^e^
**GKRH**	9.793 ± 0.485^bc^	8.902 ± 0.440^bc^	11.252 ± 0.557^d^
**GKSM**	14.490 ± 1.150^d^	13.171 ± 1.044^d^	7.624 ± 0.606^b^
**GKSH**	12.083 ± 1.775^cd^	10.985 ± 1.615^cd^	9.237 ± 1.372^c^
**ACSH**	1.330 ± 0.090^a^	1.211 ± 0.081^a^	82.829 ± 5.547^g^
**VitC**	2.710 ± 0.080^a^	2.466 ± 0.074^a^	40.577 ± 1.22^f^

Along the line, values carrying the same letter superscripts are not significantly different (P>0.05), Waller Duncan; RSA_50_, 50% Radical scavenging activity; EC_50_ = 50% Efficient Concentration; ARP, Antiradical Power; GKLM: *G*. *kola* Leaf MeOH; GKRM: *G*. *kola* roots MeOH; GKRH: *G*. *kola* roots H_2_O/EtOH; GKSM: *G*. *kola* stem MeOH, GKSH: *G*. *kola* stem H_2_O/EtOH; ACSH: *A*. *cordifolia* stem H_2_O/EtOH.

FRAP assay equally showed that, there was a significant correlation between the concentration of the extracts and their reducing power. By projecting the optical densities of the extracts on a concentration-activity curve of NH_2_OH used as standard, the results showed that ACSH generally exhibited the highest reducing power per gram of extract, followed by the hydro-ethanolic stem extract of *G*. *kola* (Table 5). At low concentrations (≤62.5 μg/mL), the activity of hydro-ethanolic stem bark extract of *A*. *cordifolia* was significantly (P≤0.05) higher than those of the other extracts, while at high concentrations (≥125 μg/mL), its activity was comparable to, at least, one of the extracts. At the maximum concentration (500 μg/mL), the ferric ion reducing capacities of all the extracts, except the methanol leave extract of *G*. *kola*, were not significantly (P>0.05) different.

**Table 5:** Quantitative evaluation of Fe^3+^ reducing power by crude extracts of *G*. *kola* and *A*. *cordifolia*.

		μg equivalent NH_2_OH/g of extract				
[Extracts] (μg/mL)	GKLM	GKRM	GKRH	GKSM	GKSH	ACSH
500	8.503 ± 2.025^eα^	11.690 ± 0.370^fᵞ^	11.563 ± 0.455^gα^	10.120 ± 0.860^eβ^	11.023 ± 0.085^gα^	11.386 ± 0.155^fβ^
250	6.133 ± 0.575^deα^	10.330 ± 0.240^eᵞ^	9.266 ± 0.125^fα^	9.743 ± 0.185^eβ^	10.873 ± 0.415^fgα^	10.990 ± 0.370^fβ^
125	7.073 ± 0.085^dα^	9.390 ± 0.870^eᵞ^	5.620 ± 0.590^eα^	5.583 ± 0.115^dβ^	10.456 ± 0.375^fα^	9.973 ± 0.215^eβ^
62.5	4.430 ± 0.320^cα^	6.193 ± 1.625^dᵞ^	3.406 ± 0.095^eα^	3.013 ± 0.165^cβ^	6.630 ± 0.350^eα^	8.773 ± 0.985^dβ^
31.25	2.596 ± 0.175^bα^	3.650 ± 0.820^cᵞ^	1.870 ± 0.030^dα^	1.816 ± 0.105^bβ^	3.893 ± 0.005^dα^	8.213 ± 0.245^dβ^
15.625	1.433 ± 0.075^abα^	1.783 ± 0.385^bᵞ^	0.996 ± 0.035^cα^	0.960 ± 0.060^aβ^	2.260 ± 0.120^cα^	5.057 ± 0.385^cβ^
7.8125	0.786 ± 0.045^aα^	0.746 ± 0.165a^bᵞ^	0.500 ± 0.020^abα^	0.566 ± 0.055^aβ^	1.196 ± 0.165^bα^	2.720 ± 0.120^bβ^

Along the line, values carrying the same letter superscripts are not significantly different (P>0.05); Across the lines, values carrying the same Greek letter are not significantly different (P>0.05), Waller Duncan; *G*KRM: *G*. *kola* roots methanol; *G*KRH: *G*. *kola* roots hydro-ethanol; *G*KSM: *G*. *kola* stem bark methanol; *G*KSH: *G*. *kola* stem bark hydro-ethanol; *G*KLM: *G*. *kola* leaves methanol; *A*CSH: *A*. *cordifolia* stem bark hydro-ethanol.

Generally, the results of the antioxidant assays showed that, the hydro-ethanolic extract presented greater antioxidant potentials than the methanolic extracts. The high antioxidant effect of the hydro-ethanolic extracts could be due to the diversity of compounds extracted by the conjugate solvent system (water/ethanol).

## Discussion

### Antibacterial activities and phytochemical compositions of extracts

The results of antimicrobial tests showed that the different parts of *G*. *kola* and the stem bark of *A*. *cordifolia* contained substances with antisalmonellal activities. The existence of these substances was confirmed by phytochemical screening which revealed the presence of certain classes of compounds (flavonoids, phenols, tannins, etc.), whose members have already been known to exhibit antimicrobial activities. According to Tamokou *et al*. [34], a plant extract is considered to be highly active if the MIC < 100 μg/mL; significantly active when 100 ≤MIC ≤512 μg/mL; moderately active when 512 <MIC ≤2048 μg/mL; weakly active if MIC > 2048 μg/mL and not active when MIC >10 000 μg/mL. Consequently, all *G*. *kola* extracts presented significant antibacterial activities (100 ≤ MIC ≤ 512 μg/mL) while moderate activities (MIC of 1000 μg/mL) were observed with the hydro-ethanolic stem bark extract of *A*. *cordifolia* (on *S*. *typhimurium* and *S*. *typhi*). It is likely that the amount of active ingredients in this plant extract does not occur in quantities large enough to produce significant activity. Such extract upon fractionation, purification and concentration of the active ingredients, may still lead to the isolation of therapeutically useful compounds against salmonellosis. However, among *G*. *kola* extracts, some of them acted on two or three *Salmonellal* species while others acted narrowly on one. Critical analysis of the composition and antisalmonellal activities of the extracts revealed that flavonoids, phenols and tannins may be responsible for the observed differences, as their mechanisms of action and/or concentration may differ. This probably explains why the methanolic root extract of *G*. *kola*, though presenting the best antisalmonellal activity, was the least complex in phytochemical groups of compounds, differing from its hydro-ethanolic extract by the presence of glycosides and terpenoids, and the absence of flavonoids in the latter, suggesting that flavonoids may play a central role in attributing activity to this plant. Contrarily, the methanolic leaf extract of *G*. *kola* and the stem bark hydro-ethanolic extract of *A*. *cordifolia* with the highest phytochemical groups of compounds showed the least antisalmonellal activity. This suggests that the presence of anthocyanins, glycosides, steroids in their extracts may have contributed to the low activity recorded, given that these were the major phytochemical groups of compounds that differentiated them from the most active. The findings of this study were in conformity with that of Flora *et al*. [35] which showed that the methanolic root and stem extracts of *G*. *kola* contained flavonoids, phenols and tanins; as well as that of Gatsing *et al*. [25] which revealed that the methanolic leaf extract did not contain alkaloids, quinones and saponins.

### Mode of action of extracts and growth kinetics

Due to the variety of bacterial targets, several models for studying the mode of antibacterial action of drugs have been described in the literature [36]. As a result, the outer membrane permeability and nucleotide leakage assays were chosen to evaluate the mode of action of the extracts against the most sensitive *Salmonella* serova (*S*. *typhi*). Apart from the hydro-ethanolic stem bark extract of *A*. *cordifolia*, all the other extracts from *G*. *kola* presented the same trend as that of Polymyxin B, indicating that they have similar modes of action, on the destabilization of the bacterial outer membrane. Looking at the percentage destabilization as a function of concentration, putting aside toxicity, the hydro-ethanolic stem extracts of *G*. *kola* could be administered at 2MIC, and those of the other *G*. *kola* extracts at MIC given that at these concentrations, their respective destabilization capacities were greater than that of Polymyxin B. The effect of these extracts on the outer membrane permeability of *S*. *typhi* could be attributed to their phytochemical contents. In effect, certain small terpenoids and phenolic compounds have been reported to disorganize and weaken the interactions between lipopolysaccharide molecules by chelating outer membrane-stabilizing divalent cations [37,38]. This antibacterial action can result in membrane expansion, increase membrane fluidity and permeability, disturbance of membrane embedded proteins, inhibition of respiration, and alterations in the ionic homeostasis between intracellular and extracellular compartments of Gram-negative bacteria [39]. This explains the significantly high destabilization effect of the terpenoid- and phenolic-rich extracts of *G*. *kola*.

Nucleotides leakage results from the damage of the cell membrane or a change in permeability of the cell membrane [40]. Various vital intracellular materials including small ions such as K^+^ and (PO_4_)^3-^ tend to leak out, followed by the leakage of large molecules such as DNA, RNA and other materials [41]. Phenolic compounds have been reported to disturb the cytoplasmic membrane, disrupt the proton motive force, electron flow and active transport [42]. Hence, they could be responsible for the observed leakage, probably triggered by the phenol-rich methanolic root extract of *G*. *kola*. Moreover, the nucleotide leakage exhibited by the extract may be associated with the presence of flavonoids. Flora *et al*. [43] attributed the antimicrobial activities of flavonoids to the disruption of microbial cells’ membranes due to their ability to chelate membrane proteins. Thus, the antibacterial flavonoids, phenols and tannins in this extract plausibly acted through the destabilization of the cell membrane.

Time-kill assays allow antibacterial agents to be classified as bacteriostatic or bactericidal and characterize the relationship between agent concentration and activity over time. The time kill profile of *G*. *kola* methanolic root extract showed that its nucleotide leakage and outer membrane permeability effects on *S*. *typhi* were bacteriostatic and concentration dependent. Bacteriostatic antimicrobial agents only inhibit the growth and multiplication of the microbes giving the immune system of the host the time to clear the microbes from the system [44]. A concentration dependent bacteriostatic effect takes place when an antimicrobial agent has a high concentration at the binding site in order to eliminate the microorganism [45]. This might explain why there was an initial increase in the number of viable cells at all concentrations over the first few hours, as the extract had to attain its binding sites before exerting its effect. This time-kill kinetics reveals that if an antisalmonellal phytomedicine is prepared from this plant, it should be administered at intervals of 8 hours (re-emergence time) following its contact with the pathogen.

### Antioxidant activities of extracts and salmonellosis related stress conditions

In addition to causing salmonellosis, it has been demonstrated that the entrance of *Salmonella* causes the production of superoxide and nitric oxide which react together to form peroxynitrite, a strong biological oxidant. This consequently increases the levels of reactive oxygen species [15]. Oxidative stress can be prevented or delayed by using antioxidant agents. Thus, having unraveled the possible antibacterial mode of action of the extracts and characterizing the relationship between their concentrations and activities over time, their antioxidant potentials were equally determined to see if in addition to their antibacterial properties, they could fight against a possible oxidative stress caused by the pathogenic invasion.

The hydro-ethanolic stem extract of *A*. *cordifolia* showed the best antioxidant potential. However, this extract, though having the best antioxidant activity, did not present significant antisalmonellal effect. This indicates that, its *Salmonella* inhibiting capacity may not be directly linked to its antioxidant activity. The extracts of *G*. *kola* presented dual (antisalmonellal and antioxidant) activities and this lends credit to their possible exploitation in the formulation of an anti-typhoid phytomedicine. The extracts could scavenge reactive oxygen species which is implicated in the pathogenesis of typhoidal and non-typhoidal *Salmonellae* [15] and therefore can fight against oxidative stress that may result from *Salmonella* infection. From the phytochemical screening and antioxidant results, the antioxidant potential of the extracts may not only be attributed to the diversity of the phytochemical groups identified, but also to the concentration of the specific group, as the methanolic leaf extract of *G*. *kola*, though being the riches in terms of phytochemical groups did not show the highest antioxidant effects. Even though most Euphorbiaceae are known to be toxic, *A*. *cordifolia* has been reported to be safe in sub-acute toxicity studies at doses ranging from 250–2000 mg/kg bw. [46]. However, the frequently used aqueous decoction of the stem barks of *G*. *kola* was shown to be toxic, with an LD_50_ value in the “very toxic” classification range (50–500mg/kg), whereas its methanol extract did not show noticeable toxicity effect; instead, the tendency of increasing erythrocyte number over time [47]. View the significant activities obtained herein, there is therefore need to do profound *in vivo* studies including toxicological analysis in the perspective of the development of a potential antityphoid phytomedicine.

## Conclusion

The results obtained in this study may thus serve as preliminary scientific data on the title species in the treatment of *Salmonella* infections in Cameroon, as they might provide good sources of active principles that could either be used as lead molecules for the synthesis of novel drugs or for a possible standardization of a phytomedicine against salmonellosis/typhoid fever. However, this could only be possible after profound *in vivo* therapeutic and toxicological studies, as well chemical profiling of the potent extract.
